# Supplementary material for: Identification and validation of a blood- based diagnostic lipidomic signature of pediatric inflammatory bowel disease
Source: Nat Commun. 2024 Jun 3;15:4567. doi: 10.1038/s41467-024-48763-7 (PMC11148148; doi:10.1038/s41467-024-48763-7)
Supplement: Supplementary file 1 — Supplementary Information [file 41467_2024_48763_MOESM1_ESM.pdf]

Supplemental Information

Title: Identification and validation of a blood-based diagnostic lipidomic signature of pediatric inflammatory bowel disease

Author(s): Samira Salihovic[1], Niklas Nyström[2], Charlotte Bache-Wiig Mathisen[3], Robert Kruse[4], Christine Olbjørn[5], Svend Andersen[6], Alexandra J Noble[7,8], Maria Dorn-Rasmussen[9,10], Igor Bazov[1], Gøri Perminow[11], Randi Opheim[3], Trond Espen Detlie[12], Gert Huppertz-Hauss[13], Charlotte R H Hedin[14,15], Marie Carlson[16], Lena Öhman[17], Maria K Magnusson[17], Åsa V Keita[18], Johan D Söderholm[18], Mauro D'Amato[19,20,21], Matej Orešič[1,22], Vibeke Wewer[9,10], Jack Satsangi[7,8], Carl Mårten Lindqvist[1], Johan Burisch[10,23], Holm H Uhlig[7,8,24], Dirk Repsilber[1], Tuulia Hyötyläinen[25]*, Marte Lie Hoivik[3]*, Jonas Halfvarson[26]*
***Shared senior authors**

Author affiliations: [1]School of Medical Sciences, Faculty of Medicine and Health, Örebro University, Örebro, Sweden; [2]Department of Women's and Children's Health, Uppsala University, Uppsala, Sweden; [3]Department of Gastroenterology, Oslo University Hospital, Oslo, Norway and Faculty of Medicine, University of Oslo, Oslo, Norway; [4]Department of Clinical Research Laboratory, Faculty of Medicine and Health, Örebro University, Örebro, Sweden; [5]Department of Pediatrics and Adolescent Medicine, Akershus University Hospital, Lørenskog, Norway; [6]Department of Pediatrics, Vestfold Hospital Trust, Tønsberg, Norway; [7]Translational Gastroenterology Unit, Nuffield Department of Experimental Medicine, University of Oxford, Oxford, United Kingdom; [8]Biomedical Research Center, University of Oxford, Oxford, United Kingdom; [9]Department of Paediatric and Adolescence Medicine, Copenhagen University Hospital - Amager and Hvidovre, Hvidovre, Denmark; [10]Copenhagen Center for Inflammatory Bowel Disease in Children, Adolescents and Adults, Copenhagen University Hospital - Amager and Hvidovre, Hvidovre, Denmark. [11]Department of Pediatric Medicine, Oslo University Hospital, Oslo, Norway; [12]Department of Gastroenterology, Akershus University Hospital, Lørenskog, Norway and Faculty of Medicine, University of Oslo, Oslo, Norway; [13]Department of Gastroenterology, Telemark Hospital Trust, Skien, Norway; [14]Karolinska Institutet, Department of Medicine Solna, Stockholm, Sweden; [15]Karolinska University Hospital, Gastroenterology unit, Department of Gastroenterology, Dermatovenereology and Rheumatology, Stockholm, Sweden; [16]Department of Medical Sciences, Uppsala University, Uppsala, Sweden; [17]Department of Microbiology and Immunology, Institute of Biomedicine, Sahlgrenska Academy, University of Gothenburg, Gothenburg, Sweden; [18]Department of Biomedical and Clinical Sciences, Linköping University, Linköping, Sweden; [19]IKERBASQUE, Basque Foundation for Science, Bilbao, Spain; [20]Gastrointestinal Genetics Lab, CIC bioGUNE - BRTA, Derio, Spain; [21]Department of Medicine & Surgery, LUM University, Casamassima, Italy; [22]Turku Bioscience Centre, University of Turku and Åbo Akademi University, Turku, Finland; [23]Gastrounit, medical division, Copenhagen University Hospital - Amager and Hvidovre, Hvidovre, Denmark; [24]Department of Paediatrics, University of Oxford, Oxford, United Kingdom; [25]School of Science and Technology, Örebro University, Örebro, Sweden; [26]Department of Gastroenterology, Faculty of Medicine and Health, Örebro University, Örebro, Sweden

***Correspondence:**
Jonas Halfvarson, Professor, Department of Gastroenterology,
Faculty of Medicine and Health, Örebro University, Örebro, Sweden.
Phone: +46 19 303000, +46 738 082361
Email: jonas.halfvarson@regionorebrolan.se

Inventory of Supporting Information

Table of Contents Page 2

Supplemental Method

Distribution of LacCer(d18:1/16:0) and PC(18:0p/22:6) in serum vs plasma Page 3

Supplementary Tables

Table S1 Page 4

Table S2 Page 5

Table S3 Page 6

Supplementary Figures

Figure S1 Page 7

Distribution of LacCer(d18:1/16:0) and PC(18:0p/22:6) in serum vs plasma

Paired serum and plasma samples from healthy volunteers (N=5) were analyzed to compare LacCer(d18:1/16:0) and PC(18:0p/22:6) concentrations using the described methodology. Results revealed no significant differences between serum and plasma concentrations for both LacCer(d18:1/16:0) (369 ng/mL in serum vs 339 ng/mL in plasma, $P = 0.13$) and PC(18:0p/22:6) (223 ng/mL in serum vs 218 ng/mL in plasma, $P = 0.72$). These findings indicate no significant differences between paired plasma and serum concentrations.

Table S1. Discovery cohort (N=94) modelling specifics and validation cohort performance of evaluated machine learning algorithms on 169 lipids with and without prior feature selection for diagnostic prediction of pediatric IBD *vs* symptomatic controls.

**Algorithm method**	**R package**	**Hyperparameters tuned**	**Averaged AUC of models on all lipids (95% CI)**	**Averaged AUC of models following Boruta selection of lipids (95% CI)**
Penalized regularized logistic regressions (LR)	glmnet	mixture and penalty	0.76 (0.73-0.79)	0.81 (0.79-0.84)
Random forests (RF)	ranger	number of variables included in each random tree and minimum n for split	0.81 (0.78-0.83)	0.78 (0.76-0.81)
XGBoosted trees (XG)	xgboost	number of variables included in each tree, tree depth, loss reduction, learning rate, and minimum n for split	0.76 (0.72-0.79)	0.78 (0.75-0.81)
Neural network (NN)	nnet	number of hidden layers and penalty	0.76 (0.73-0.79)	0.79 (0.76-0.82)
Naïve Bayes (NB)	klaR	smoothness and laplace	0.63 (0.60-0.66)	0.70 (0.67-0.74)
LightGBM	bonsai	number of variables included in each tree, tree depth, loss reduction and learn rate	0.79 (0.76-0.82)	0.75 (0.72-0.78)
Radial basis support vector machine (SVM)	discrim	cost and sigma	0.70 (0.66-0.73)	0.74 (0.71-0.77)
Stacked model of LR, RF and NN (ST)	stacks	Individually for LR, RF and NN as above	0.79 (0.79-0.82)	0.82 (0.79-0.84)

Discovery cohort training and validation cohort testing procedure:

The discovery cohort was partitioned into training (75%) and internal testing (25%) sets. The variables of the training set were either used as a full set of all 169 lipids or reduced by Boruta selection with a cut-off of 0.01 (boruta package in R). Each set of data was standardized, and bagged tree model imputation of missing data (recipe package in R) and class balancing with Adaptive Synthetic Algorithm minority over-sampling technique (themis package in R) was performed on the training set. Tuning was performed with a Latin Hypercube search approach with internal validation on 15 bootstraps of the training data with classification performance evaluation scored as AUC on the discovery cohort testing set. Optimal hyperparameters were used in the final models and performance was assessed with the validation cohort data. This procedure was repeated iteratively 10 times as randomized nested cross-validation with random partitioning of the discovery cohort to training and testing set at each iteration to estimate the robustness of predictions with regards to random effects of discovery cohort partitioning. Area under the receiver operating curves (AUCs) averaged over nests are presented in the table.

Table S2. Youden index, sensitivity, specificity, positive likelihood ratio (LR), and negative LR of a signature of hsCRP, LacCer(d18:1/16:0) and PC(18:0p/22:6), and fecal calprotectin in predicting pediatric IBD in the validation cohort among children and adolescents who provided fecal samples (N=77).

Evaluated model	Youden Index (J)	Sensitivity (%)	Specificity (%)	LR(+)	LR(-)
hsCRP	0.50	70.2	75.0	2.8	0.4
PC(18:0p/22:6) and LacCer(d18:1/16:0)	0.73	82.5	90.0	8.2	0.2
Fecal calprotectin	0.75	82.5	90.0	8.2	0.2

Abbreviations: hsCRP, high sensitivity C-reactive protein; LR(+), likelihood ratio for positive test result; LR(-), likelihood ratio for a negative test result

Table S3. Demographics and clinical characteristics of the pediatric confirmation cohort.

	IBD		Symptomatic controls*
	Crohn's disease	Ulcerative colitis	
N	110	54	99
Median age, years (IQR 1-3)	14 (11-16)	14 (13-16)	10 (6-14)
Males, n (%)	61 (55)	27 (50)	55 (56)
Location of CD, n (%)			
L1 (terminal ileum)	11 (10)		
L2 (colon)	27 (25)		
L3 (ileocolon)	35 (32)		
L4 A* (upper GI)	0 (0)		
L4 B** (upper GI)	2 (2)		
Lx (unknown)	35 (32)		
Behavior of CD, n (%)			
B1 (non-stricturing, non-penetrating)	76 (69)		
B2 (stricturing)	6 (5)		
B3 (penetrating)	9 (8)		
Bx (unknown)	12 (11)		
p (perianal disease)	7 (6)		
Extent of UC, n (%)			
E1 (proctitis)		10 (19)	
E2 (left sided)		10 (19)	
E3 (extensive)		9 (17)	
E4 (pancolitis)		13 (24)	
Ex (unknown)		12 (22)	

*Out of 99 symptomatic controls, 30 were diagnosed with celiac disease.

Abbreviations: IBD, inflammatory bowel disease; CD, Crohn’s disease; UC, ulcerative colitis; GI, gastrointestinal.

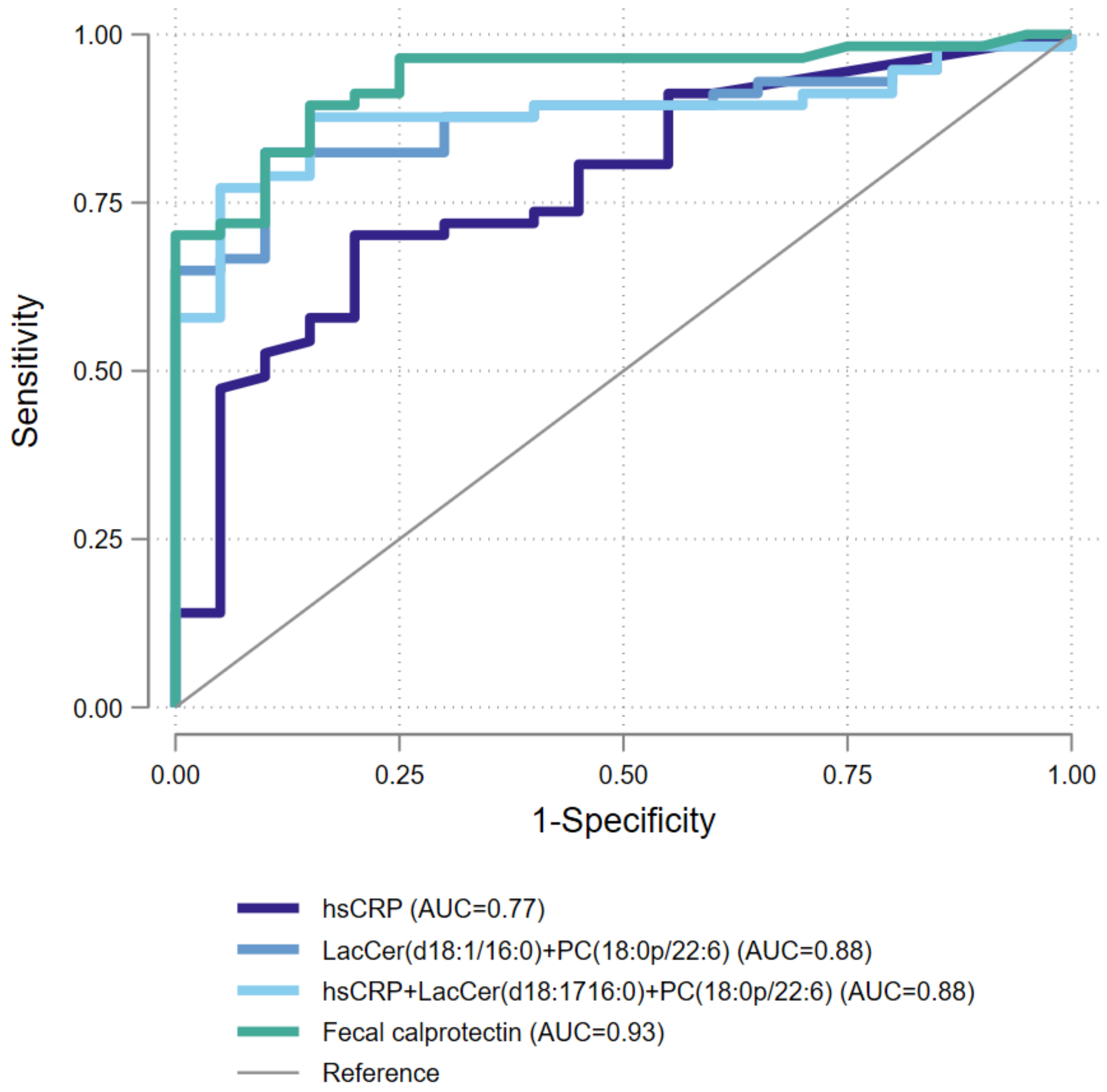

Figure S1. Receiver operating curve (ROC) for diagnostic prediction of pediatric IBD among the 77 out of 117 children that provided fecal samples in the validation cohort using logistic regression. Model performance and validity measures were as follows: Area under the curve (AUC) for hsCRP was 0.77 (95% CI 0.65-0.89), AUC for the top two validated molecular lipid species LacCer(d18:1/16:0) and PC(18:0p/22:6) was 0.88 (95% CI 0.80-0.95), AUC for high-sensitivity C-reactive protein (hsCRP) in combination with the two top validated lipids was 0.88 (95% CI 0.80-0.95), and finally the AUC for fecal calprotectin was 0.93 (95% CI 0.88-0.99).
