# Supplementary material for: Identification and validation of a blood- based diagnostic lipidomic signature of pediatric inflammatory bowel disease
Source: Nat Commun. 2024 Jun 3;15:4567. doi: 10.1038/s41467-024-48763-7 (PMC11148148; doi:10.1038/s41467-024-48763-7)
Supplement: Supplementary file 3 — Description of Additional Supplementary Files [file 41467_2024_48763_MOESM3_ESM.pdf]

Description of Additional Supplementary Files

File Name: Supplementary Data 1.

Description: Table providing the receiver operating characteristic (ROC) area under the curves (AUCs) of the diagnostic prediction of pediatric inflammatory bowel disease (IBD) in the validation cohort using logistic regression.

File Name: Supplementary Data 2.

Description: Table providing the receiver operating characteristic (ROC) area under the curves (AUCs) of the diagnostic prediction of pediatric inflammatory bowel disease (IBD) in the validation cohort among the patients who provided stool samples (N=77) using logistic regression.

File Name: Supplementary Data 3.

Description: Table providing the pair-wise correlations of age, body mass index (BMI), high-sensitivity C-reactive protein (hsCRP), albumin, fecal calprotectin, lactosyl ceramide (LacCer) d18:1/16:0 and phosphatidylcholine (PC) 18:0p/22:6 among all participants in the discovery cohort were assessed using Pearsons correlation coefficient.

File Name: Supplementary Data 4.

Description: Table showing estimated beta coefficients, standard error, 95% confidence intervals, and P-values for the logistic regression of lactosyl ceramide (LacCer) d18:1/16:0 and inflammatory bowel disease when introducing age and body mass index (BMI) as interaction terms in the validation cohort (N=117).

File Name: Supplementary Data 5.

Description: Table showing estimated beta coefficients, standard error, 95% confidence intervals, and P-values for the logistic regression of phosphatidylcholine (PC) 18:0p/22:6 and inflammatory bowel disease when introducing age and body mass index (BMI) as interaction terms in the validation cohort (N=117).
