# Supplementary material for: Identification and validation of a blood- based diagnostic lipidomic signature of pediatric inflammatory bowel disease
Source: Nat Commun. 2024 Jun 3;15:4567. doi: 10.1038/s41467-024-48763-7 (PMC11148148; doi:10.1038/s41467-024-48763-7)
Supplement: Supplementary file 9 — Reporting Summary [file 41467_2024_48763_MOESM9_ESM.pdf]

nature portfolio

Corresponding author(s): Jonas Halfvarson

Last updated by author(s): Feb 29, 2024

Reporting Summary

Nature Portfolio wishes to improve the reproducibility of the work that we publish. This form provides structure for consistency and transparency in reporting. For further information on Nature Portfolio policies, see our Editorial Policies and the Editorial Policy Checklist.

Statistics

For all statistical analyses, confirm that the following items are present in the figure legend, table legend, main text, or Methods section.

n/a	Confirmed	
☐	☒	The exact sample size (n) for each experimental group/condition, given as a discrete number and unit of measurement
☐	☒	A statement on whether measurements were taken from distinct samples or whether the same sample was measured repeatedly
☐	☒	The statistical test(s) used AND whether they are one- or two-sided<br>*Only common tests should be described solely by name; describe more complex techniques in the Methods section.*
☐	☒	A description of all covariates tested
☐	☒	A description of any assumptions or corrections, such as tests of normality and adjustment for multiple comparisons
☐	☒	A full description of the statistical parameters including central tendency (e.g. means) or other basic estimates (e.g. regression coefficient) AND variation (e.g. standard deviation) or associated estimates of uncertainty (e.g. confidence intervals)
☐	☒	For null hypothesis testing, the test statistic (e.g. F, t, r) with confidence intervals, effect sizes, degrees of freedom and P value noted<br>*Give P values as exact values whenever suitable.*
☒	☐	For Bayesian analysis, information on the choice of priors and Markov chain Monte Carlo settings
☒	☐	For hierarchical and complex designs, identification of the appropriate level for tests and full reporting of outcomes
☐	☒	Estimates of effect sizes (e.g. Cohen's d, Pearson's r), indicating how they were calculated

Our web collection on statistics for biologists contains articles on many of the points above.

Software and code

Policy information about availability of computer code

Data collection	NA
Data analysis	Statistical analyses were performed using the statistical computing language STATA16 and R (version 4.1.2).

For manuscripts utilizing custom algorithms or software that are central to the research but not yet described in published literature, software must be made available to editors and reviewers. We strongly encourage code deposition in a community repository (e.g. GitHub). See the Nature Portfolio guidelines for submitting code & software for further information.

Data

Policy information about availability of data

All manuscripts must include a data availability statement. This statement should provide the following information, where applicable:

- Accession codes, unique identifiers, or web links for publicly available datasets
- A description of any restrictions on data availability
- For clinical datasets or third party data, please ensure that the statement adheres to our policy

Lipidomics data have been deposited to the EMBL-EBI MetaboLights database with the identifier MTBLS9668 and the complete dataset can be accessed via https://www.ebi.ac.uk/metabolights/MTBLS9668. The clinical data associated with the lipidomics dataset are not publicly available because of patient confidentiality. However, the data can be made available for IBD research upon request through a minimal access procedure. This procedure consists of sending a request per email to the corresponding author (Jonas Halfvarson, jonas.halfvarsson@regionorebrolan.se) including a copy of the ethics approval. A response will be provided

within two weeks. This procedure is installed to ensure that the clinical data are being requested for scientific purposes only and thus complies with the informed consent signed by the participants, since the collected data cannot be used by commercial parties. Source data are provided with this paper.

Research involving human participants, their data, or biological material

Policy information about studies with human participants or human data. See also policy information about sex, gender (identity/presentation), and sexual orientation and race, ethnicity and racism.

Reporting on sex and gender	Information about sex is provided, and sex is used as a covariate in the analyses.
Reporting on race, ethnicity, or other socially relevant groupings	No race, ethnicity, or other socially relevant variables were used in the study.
Population characteristics	Covariate-relevant population characteristics included age, sex, and BMI, as well as clinical characteristics of inflammatory bowel disease. Basic demographics and clinical characteristics are reported.
Recruitment	Incident pediatric patients were prospectively recruited in two independent pediatric inception IBD cohorts, i.e. the discovery- and validation cohort. In the confirmation cohort, Danish and Norwegian pediatric patients were prospectively included, whereas prevalent cases and controls were recruited in the UK.
Ethics oversight	Ethical permission was granted by the Uppsala University Ethics Committee, Sweden (2008/395), the South Eastern regional Ethical board, Norway (2015/946), the Ethics Committee of the Capital Region of Denmark (H-20065831), the Danish Data Protection Agency (P-2020-1065), and the Oxford Research Ethics Committee, Reference: 11/YH/0020 and 16/YH/0247.

Note that full information on the approval of the study protocol must also be provided in the manuscript.

Field-specific reporting

Please select the one below that is the best fit for your research. If you are not sure, read the appropriate sections before making your selection.

☒ Life sciences ☐ Behavioural & social sciences ☐ Ecological, evolutionary & environmental sciences

For a reference copy of the document with all sections, see nature.com/documents/nr-reporting-summary-flat.pdf

Life sciences study design

All studies must disclose on these points even when the disclosure is negative.

Sample size	Incident patients were prospectively recruited in the population-based discovery and validation cohorts during the study period. Therefore, the number of included patients was not pre-defined by power calculations. However, results from the discovery cohort was validated in the validation cohort and, ultimately also in the confirmation cohort.
Data exclusions	No data were excluded.
Replication	Two independent pediatric inception IBD cohorts were examined. Samples were prospectively collected before the initiation of treatment and results from the discovery cohort were replicated in the validation cohort. Moreover, we performed targeted analyses and absolute quantifications of the identified molecular lipids in an independent third cohort, i.e. the confirmation cohort.
Randomization	NA
Blinding	The investigators were blinded to group allocation during the chemical analyses.

Reporting for specific materials, systems and methods

We require information from authors about some types of materials, experimental systems and methods used in many studies. Here, indicate whether each material, system or method listed is relevant to your study. If you are not sure if a list item applies to your research, read the appropriate section before selecting a response.

Materials & experimental systems

n/a	Involved in the study
☒	☐ Antibodies
☒	☐ Eukaryotic cell lines
☒	☐ Palaeontology and archaeology
☒	☐ Animals and other organisms
☐	☒ Clinical data
☒	☐ Dual use research of concern
☒	☐ Plants

Methods

n/a	Involved in the study
☒	☐ ChIP-seq
☒	☐ Flow cytometry
☒	☐ MRI-based neuroimaging

Clinical data

Policy information about clinical studies
All manuscripts should comply with the ICMJE guidelines for publication of clinical research and a completed CONSORT checklist must be included with all submissions.

Clinical trial registration: NA

Study protocol: As this is an observational study, with recruitment initiated many years ago, no separate study protocol for this research has been published prior to this submission, but a copy of the protocol and the statistical analysis plan has been uploaded on the portal.

Data collection: The discovery cohort consisted of pediatric patients aged <18 years with suspected IBD who were referred to the Uppsala University Children´s hospital between 2009 and 2018. The validation cohort included patients aged <18 years from IBSEN III, a population-based inception cohort from a geographically well-defined area, i.e. the Norwegian South-Eastern Health Region in Norway. All patients in the validation cohrot were included from 2017-2019. In the confirmation cohort, pediatric patients were included in Denmark 2021-2023, Norway 2009-2012, and the UK 2011-2017.

Outcomes: The diagnosis of IBD was based on the ESPGHAN/Porto criteria.

Plants

Seed stocks: NA

Novel plant genotypes: NA

Authentication: NA
